# Spatially Ordered Arrays of Colloidal Inorganic Metal Halide Perovskite Nanocrystals via Controlled Droplet Evaporation in a Confined Geometry

**DOI:** 10.3390/ma14226824

**Published:** 2021-11-12

**Authors:** Kwan Lee, Jonghyun Moon, Jeonghwa Jeong, Suck Won Hong

**Affiliations:** 1 Department of Advanced Materials Engineering, Kyungsung University, Busan 48434, Korea; 2 Department of Cogno-Mechatronics Engineering, Department of Optics and Mechatronics Engineering, Pusan National University, Busan 46241, Korea; moonticle@gmail.com (J.M.); 2jeong.s.o@gmail.com (J.J.)

**Keywords:** perovskite QD, self-assembly, coffee-ring effect, contact line

## Abstract

Inorganic metal halide perovskite nanocrystals, such as quantum dots (QDs), have emerged as intriguing building blocks for miniaturized light-emitting and optoelectronic devices. Although conventional lithographic approaches and printing techniques allow for discrete patterning at the micro/nanoscale, it is still important to utilize intrinsic QDs with the concomitant retaining of physical and chemical stability during the fabrication process. Here, we report a simple strategy for the evaporative self-assembly to produce highly ordered structures of CsPbBr_3_ and CsPbI_3_ QDs on a substrate in a precisely controllable manner by using a capillary-bridged restrict geometry. Quantum confined CsPbBr_3_ and CsPbI_3_ nanocrystals, synthesized via a modified hot-injection method with excess halide ions condition, were readily adapted to prepare colloidal QD solutions. Subsequently, the spatially patterned arrays of the perovskite QD rings were crafted in a confirmed geometry with high fidelity by spontaneous solvent evaporation. These self-organized concentric rings were systemically characterized regarding the center-to-center distance, width, and height of the patterns. Our results not only facilitate a fundamental understanding of assembly in the perovskite QDs to enable the solution-printing process but also provide a simple route for offering promising practical applications in optoelectronics.

## 1. Introduction

Owing to their superior physical properties, inorganic metal halide perovskite nanocrystals have attracted great attention in the field of photonics and optoelectronics for a variety of applications, including photovoltaic cells, light-emitting diode (LED)-based displays, photodetectors, and lasers [1,2,3,4,5]. By developing integration techniques onto the electronic systems, the various types of perovskite materials have been continuously produced in a simple synthetic manner with highly resolved structures, such as quantum dots (QDs), representing a quantized photoluminescence emission of a wide spectrum [6,7,8,9]. From a fundamental point of view, the perovskite thin films can be grown on a crystalline substrate by a firmly established method, such as molecular beam epitaxy (MBE) [10]. However, the process has some drawbacks, especially the lack of controllability in scale-up fabrication. On the other hand, colloidal semiconductor QDs enable an imprinting process for mass production over a large area without the phase transition from solution to solid state [11]. Such an accurately programmable solution-based technique is preferred to satisfy reproducibility in pattern formation. Thus, the high light-emitting efficiency with an extended lifetime can be the main issue to take advantage of QDs with the concomitant retaining of physical and chemical stabilities [12,13,14]. The high uniformity and low dispersity of perovskite QDs in an appropriate solvent are critically required in a colloidal form [13,14].

Therefore, in practical use, the main goal is to assemble them into organized super-structures, such as uniform thin films or patterned arrays, with a controlled manufacturing process. In this concern, conventional lithographic approaches can be adapted to realize discrete pattern formation in the micro/nanoscale [15,16]. However, the intrinsic feature of instabilities in the process generally leads to degradation for the inorganic metal halide perovskite nanocrystals during the consecutive multiple steps, including etching and templating stages [17,18]. Thus, other alternative methods have been suggested and processed with multi-dimensions. In this regard, self-assembly of perovskite QDs can be one powerful option to form highly ordered structures, minimizing the randomly organized dissipative structure formation [19]. Assemblies and controlled positioning of perovskite QDs are important to represent collective properties, which highly depend on the size, shape, and surface chemistries. Recent advances in self-assembly techniques have witnessed versatile opportunities to create some miniaturized optoelectronic devices by employing external fields [20,21,22]. For example, by a preparation of the nanoscale confinement geometry, evaporative self-assembly of nanoparticles has been demonstrated for fabricating an array of defined patterned structures, which was possibly associated with the transfer printing of the nanoscale colloidal templates [23]. Similarly, a confined structure with a defined hole-array with vertical microfluidic channels produced by a standard lithographic Si wafer etching process enabled the deposition of colloidal nanoparticles in a planar configuration [24]. With the progress of the integration technique for the solution processible method, a sophisticated toolkit (i.e., ink-jet printing) was ready to apply for a small-scale deposition of nanoparticles or conductive organic molecules; this computer-controlled positioning of colloidal solution through multiple nozzles is highly promising for a variety of optoelectronic applications [25]. However, the above set of experimental approaches still required other expensive equipment or a series of processing steps. On the other hand, as a simple and rapid one-step process, evaporative self-assembly of nonvolatile solutes from a single droplet on a substrate to realize repetitive patterns consisting of nanomaterials has represented high yield in the spatial arrangement of the nanomaterials [26]. Evaporation of a drop containing nonvolatile solutes on a single surface enables the formation of “*coffee-ring*”, where the evaporation rate is maximized at the edge of a sessile droplet of the solution with a pinned contact line as the solvent evaporates [27,28,29]. The pinned contact line allows for the transportation of solutes to the extremity of the droplet. By changing the solution concentration, the multiple rings are spontaneously formed. However, the resulted surface patterns are found to be stochastic patterns without controllable regularity because that phenomenon is far from the equilibrium state in the solvent evaporation process [30]. Thus, unique confined geometries were developed to control the evaporation flux and interfacial interaction between the depositing solutes and the substrate. Recently, to solve this problem, a representative sphere-on-flat geometry was implemented to control the hydrodynamic flow in the microfluidic situation [26,31,32], in which the competition between the capillary and the pinning forces was regulated. This represented a strategic solution to produce tailored nanostructures with high fidelity and regularity for useful devices and demonstrated an unconventional lithographic strategy by simple solvent evaporation [33].

Here, we report a facile technique to generate multiple pattern formation over a defined area by utilizing evaporative self-assembly in confined geometry using a perovskite QD solution. A droplet of QD solution was naturally evaporated in a geometry bridged with an upper convex lens and lower flat SiO_2_/Si substrate. During evaporation in a stationary condition, the cyclic pinning/depinning motions of the contact line spontaneously left behind highly ordered structures consisting of perovskite QDs. Optically, these periodic micro/nanoscale patterns were light-emissive under UV excitation concerning their intrinsic structures. We focused on the material system of CsPbX_3_ that was synthesized through a modified hot-injection approach following the previous method [34]. Two types of nanocrystals were selected as nonvolatile solutes for the pattern formation (i.e., CsPbBr_3_ and CsPbI_3_, photoluminescent emission of 495 and 647 nm, respectively). We believe that our strategy in manipulating the patterning process is easy to implement for the effective spatial arrangement of the nanomaterials of interest, such as metal halide perovskite QDs and other small molecules, for widespread applications in optoelectronic devices [35].

## 2. Materials and methods

### 2.1. Materials

All chemicals were used as received: lead bromide (Aldrich, St. Louis, MO, USA, ≥99.9%), lead iodide (Aldrich, ≥97%), cesium carbonate (Aldrich, ≥99.9%), zinc bromide (Aldrich, ≥99.9%), zinc iodide (Aldrich, ≥99.9%), oleic acid (Aldrich, technical grade 90%), oleylamine (Aldrich, 70%), 1-octadecene (Aldrich, 90%), anhydrous hexane, and anhydrous toluene.

### 2.2. Synthesis of CsPbBr_3_ and CsPbI_3_

Preparation of Cs-oleate precursor solution, Cs_2_CO_3_ (0.5 mmol), in a mixture of oleic acid (0.5 mL) and 1-octadecene (8 mL), was dissolved at 150 °C for 60 min under nitrogen atmosphere.

CsPbBr_3_ and CsPbI_3_ nanocrystals were prepared through a hot-injection approach by the previous report, with some modifications [34]. PbX_2_ (75 mg of PbBr_2_ and 120 mg of PbI_2_) was dissolved with 300 mg of ZnX_2_ in a mixture of 1-octadecene (10 mL), oleic acid (1 mL), and oleylamine (1 mL) in a 50 mL three-neck round-bottomed flask under a nitrogen atmosphere at 120 °C for 60 min. After drying, the solution was heated up and kept at 160 °C. The Cs-oleate solution (0.8 mL) was rapidly injected into the reaction flask, in which the Cs-oleate solution was maintained at 160 °C. The reaction was kept for 10 s and then the crude solution was cooled in an ice bath. The crude solution was diluted with anhydrous hexane and centrifuged at 3000 rpm to remove the precipitate of the unreacted slats. The nanocrystals dispersed in the supernatant were collected under 10,000 rpm for 10 min. The purification was repeated 3 times to remove the unreacted salts precipitate and undesired side products. The precipitated nanocrystals redispersed in anhydrous toluene were used for further characterization.

### 2.3. Self-Assembly of the Colloidal Perovskite QD Solutions in Confined Geometry

A spherical silica lens was placed on the SiO_2_/Si or glass substrate in a sealed chamber to control and monitor the inside environment, including temperature and humidity. Each CsPbBr_3_ and CsPbI_3_ QDs colloidal toluene solution was loaded in a gap between the spherical lens and a flat substrate (i.e., sphere-on-flat geometry). The controllable gap distance of two surface gaps enables it to form a capillary bridge of the perovskite toluene solution (c = 0.2 mg mL^−1^). The sphere-on-flat geometry was allowed to evaporate the solution; complete evaporation of a droplet (i.e., 15 μL) took 40 min in this work. After drying, the spherical lens was carefully separated from the substrate, and the results were characterized. A typical relative humidity (RH) in this experiment was monitored at the range of ~20–30% in a sealed chamber.

### 2.4. Characterization

The synthesized perovskite CsPbBr_3_ and CsPbI_3_ QDs were characterized with transmission electron microscopy (Hitachi, H-7500, Tokyo, Japan) to confirm their shape and morphology. The optical properties of the perovskite QDs were estimated regarding absorption (Shimadzu, Kyoto, Japan, UV-1800) and photoluminescence (Shimadzu, RF-5301PC). The ring patterns were examined regarding pattern structures, the center-to-center distance, the height, and the width with optical microscopy (Olympus BX51, Ulsan, Korea), scanning electron microscopy (SEM, Hitachi S-4800, Tokyo, Japan), and atomic force microscopy (AFM, Park systems NX20, Tokyo, Japan) devices.

## 3. Results and Discussion

**Figure 1:**
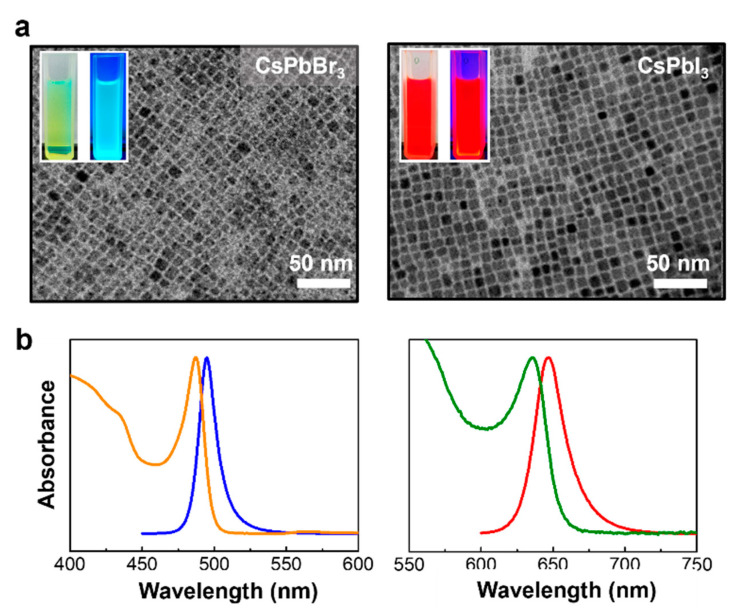
Inorganic metal halide perovskite QDs via hot-injection approach with zinc halide salt at 160 °C. (**a**) Typical TEM images of CsPbBr_3_ and CsPbI_3_ QDs synthesized using ZnBr_2_ and ZnI_2_ as an excess of halide ions, respectively. Insets show digital images of CsPbBr_3_ and CsPbI_3_ QDs dispersed in toluene solvent, which is illuminated under irradiation with an excitation wavelength of 365 nm. (**b**) Absorption (orange line of CsPbBr_3_ and green line for CsPbI_3_ QDs) and corresponding PL spectra (blue line of CsPbBr_3_ and red line of CsPbI_3_ QDs) shows emission center of 495 and 647 nm, respectively; excitation wavelength was 350 nm.

The separation between nucleation and growth is the typical approach to tune the electronic bandgap of semiconductor nanocrystals, in which the tailored size allows the quantum confinement of excitons. However, the rapid growth through hot-injection methods for inorganic metal halide perovskite nanocrystals made it difficult to kinetically control their dimension with high uniformity. In these concerns, tailoring halide ions equilibrium between the nanocrystals lattice and surrounding solvent medium led to the size control of CsPbX_3_ quantum dots [34,36]. It is worth noting that a halide-rich surface through the equilibrium of halide allows for a decrease in the size of QDs when synthesized with excess halide in the reactant mixture [34]. As presented in Figure 1, the CsPbBr_3_ and CsPbI_3_ QDs were prepared separately with an optimized concentration of ZnBr_2_ and ZnI_2_ reagents to provide excess halide from the zinc salts and excess zinc ions remained as an inert bystander. Figure 1a shows the transmission electron microscopy (TEM) images of the regular cubic arrangement of CsPbBr_3_ and CsPbI_3_ QDs due to their uniform size and shape. The QDs were synthesized under an optimized metal halide concentration (1 mmol of ZnBr_2_ and ZnI_2_) with lead halide reagents at 160 °C via hot-injection of Cs-oleate precursor. The structure morphology of TEM images displays the cubic CsPbBr_3_ and CsPbI_3_ QDs with an edge length of 7.21 and 7.58 nm, respectively (variation coefficient less than 16%, Supporting Information Figure S1). The Bohr radius for each CsPbBr_3_ and CsPbI_3_ QD was estimated to be 7 and 12 nm, respectively [2]. The quantum confinement of the prepared QDs was evaluated by the optical spectra of absorption and photoluminescence (PL) measurement, as shown in Figure 1b. The blue shift of the well-resolved absorption peaks (495 nm for CsPbBr_3_ QDs, and 647 nm for CsPbI_3_ QDs) from the band edge exciton were exhibited. Our observation shows that both rapid cooling to room temperature using iced water and excess halide ions from zinc salt allowed for the disintegration of the QDs, in which low temperature by cooling and halide equilibrium with extra halide ions provided an energy barrier to maintain the stability of the QDs [34,36]. An integration of the QDs and morphology change without either rapid cooling or excess zinc salts was observed as shown in Figure S2.

**Figure 2:**
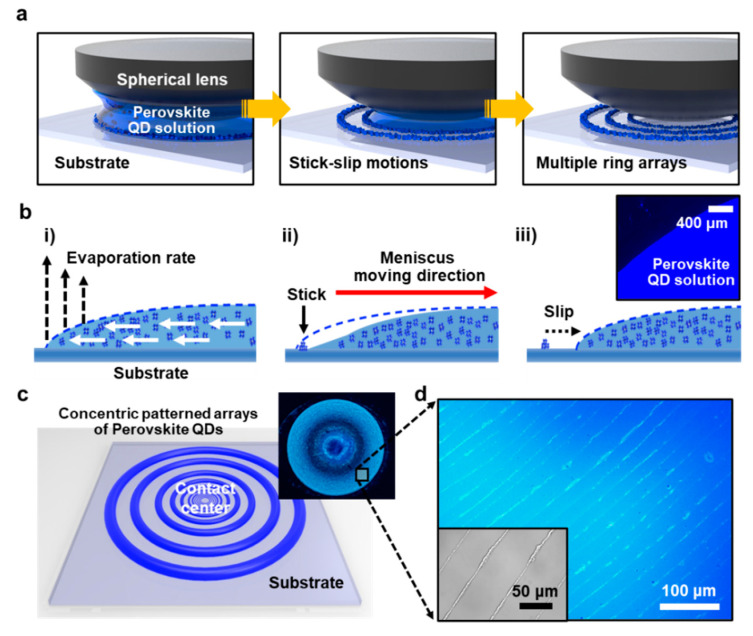
Evaporative self-assembly of the perovskite QDs in a sphere-on-flat geometry. (**a**) Stepwise schematics of the formation of the multiple ring arrays via drying-mediated assembly process of colloidal perovskite QDs. A sphere-on-flat geometry forms a capillary-held solution with the maximum evaporative loss of solvent at the capillary edge, which leads to repetitive stick-slip motions. (**b**) Cross-sectional demonstration of a perovskite QD ring formation by a stick-slip event. The dotted line represents a change of both the contact angle of the colloidal QDs solution and the solution volume of the meniscus. (**c**) Schematic of a concentric patterned array of the perovskite QD rings after the complete solvent evaporation and a digital image of CsPbBr_3_ QD multiple ring arrays under UV irradiation. (**d**) A representative optical micrograph of highly ordered perovskite QD rings formed on a SiO_2_/Si substrate. The inset image shows a phase-contrast image for tiny stripes of CsPbBr_3_ on a glass substrate.

Figure 2a sequentially illustrates the pattern formation process on the concentric multiple rings, crafted by the consecutive pinning/depinning contact lines of perovskite QDs. At the initial stage of the experiment (left panel in Figure 2a), the QDs dispersed in anhydrous toluene with an optimized concentration (i.e., *c* = 0.2 mg mL^−1^) were trapped between the spherical lens (fused silica: ~10 mm in diameter, radius curvature: 2.7 mm) and the flat SiO_2_/Si substrate, in which the gap distance was controlled by the micropositioning stage and fully contacted to form a capillary bridge. After the capillary-held QD solution was introduced, the solvent evaporation proceeded in a sealed chamber in this unique geometry, where the restricted evaporation rate was induced only on the edge of the droplet. Thus, the evaporation rate was restricted and controlled until the final stage of the evaporative self-assembly. This experimental scheme is different from the typical process for a single ring-pattern formation, known as the coffee-ring effect, representing an engineered approach to facilitate stick-slip motions to manipulate multiple ring arrays (right panel in Figure 2a). The detailed illustration in Figure 2b describes a consecutive stick-slip motion of the moving meniscus. Within the confined geometry, once the pinning site is provided at the edge of the contact line (i.e., stick), the contact angle is gradually decreased due to the continuous solvent evaporation. When the meniscus slowly moves to the direction of the contact-center (Figure 2b(ii)), the perovskite QDs are jammed at the contact line with the balanced surface tension in a certain period (i.e., ring formation). At this moment, the pattern can be formed by continuous deposition of the colloidal perovskite QDs. Then, as the inward capillary force reaches a higher level than the pinning force, the pinned meniscus quickly moves to the new position by a rapid recession of the contact line, recovering the initial contact angle with replenished solutes from the internal solution (i.e., slip, Figure 2b(iii)). This repetitive stick-slip motion of the moving meniscus leaves behind multiple ring arrays on the substrate. In other words, a concentrated evaporative loss of the dropped solvent at the contact lines can lead to the flow of the suspended solutes to the multiple pinning sites and induced periodic deposition of the solutes. It should be acknowledged that this unique geometry enables the maximum evaporative loss only at the capillary edge on the substrate by blocking the solvent evaporation at the center region of the lens, at which the circular shape of the droplet was also maintained following the lens, and thus perfect ring-patterns can be produced. Notably, the upper surface (i.e., spherical lens) directed the evaporation of solvent at the contact lines, defining the well-ordered nanostructure arrays of perovskite QDs, unlike the irregular and unreliable pattern formation typically observed in single droplet experiments [31].

During the initially performed control experiments, the purification and concentration range was monitored to qualify the colloidal QD solutions in our confined geometry, as shown in Figure S3. Since the delicate interaction within the two surfaces is the main parameter, the careful purification of the perovskite QDs was found to be important in the same optimized concentration range (*c* = 0.2 mg mL^−1^) as shown in Figure S3a. In addition, the environmental temperature was also critical to maintaining stable stick-slip motions in the process. When the slightly elevated temperature was provided in the chamber, chaotic dissipated patterns were observed as shown in Figure S3b,c; these results may have originated from an unwanted Marangoni effect inducing local convection flow in the trapped droplet [37]. By changing the concentration of the lower level (i.e., *c* = 0.1 mg mL^−1^), tiny lines but disconnected ring patterns with fingering instabilities were observed (Figure S3d). However, under an optimized condition, complete evaporation within the geometry under a controlled condition finally allowed for the spontaneous discrete patterns with unprecedented regularity on the flat substrate at the temperature range of ~25–40 °C [38].

Figure 2c schematically demonstrates the concentric patterned array of perovskite QDs in a gradient fashion from the outermost to the innermost region. The inset digital image shows blue emission from the finite ring-patterned arrays of CsPbBr3 on the SiO_2_/Si flat surface under the excitation of a UV lamp (365 nm wavelength). In this experiment, a small drop of CsPbBr_3_ colloidal solution (15 μL) was used by trapping it in a sphere-on-flat geometry, and the diameter of the concentric ring at the outermost region was ~8 mm. The patterned arrays of the perovskite QDs were observed by optical microscope as shown in Figure 2d, where the measured area represents highly ordered, macroscopic, discretely patterned line-spacing stripes. In some locations, the patterned arrays of the perovskite QDs displayed dimensional variations with surface perturbation compared to the previous reports, which may be attributed to the local condensation of solute and the “solutal Marangoni effect” [39]. The inset shows a phase-contrast image for tiny stripes of CsPbBr_3_ on a glass substrate. This implies that the pattern formation of the perovskite QDs was not limited to the selection of the substrate such as glass, ITO, metals, or Si.

**Figure 3:**
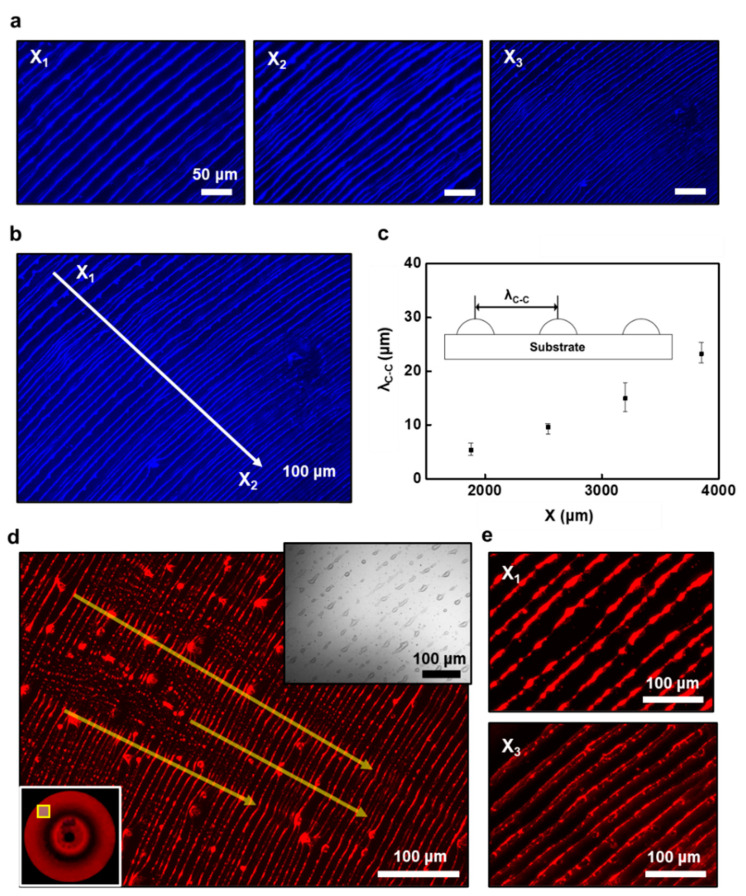
Multiple ring arrays of CsPbBr_3_ and CsPbI_3_ QDs. (**a**) Fluorescence microscopic images from three different positions (*X*_1_, *X*_2_, and *X*_3_ from outermost to innermost regions) of the patterned arrays of CsPbBr_3_ QD rings; highly dense arrays of the rings with blue-emission under the fluorescence. (**b**) A typical microscopic image shows the finite aligned arrays structure of the CsPbBr_3_ QDs with a finely tuned controllability. (**c**) A center-to-center distance (λ_c-c_) of the radially formed CsPbBr_3_ QD rings plotted as a distance from the contact-center shows a gradient fashion. (**d**) Typical optical micrograph of the patterned array of CsPbI_3_ QD ring with red-emission under the fluorescence. (**e**) Highly magnified optical micrographs with discretely patterned arrays of QD rings in a gradient trend at the outermost (upper) and innermost regions (lower).

To observe the spatial arrangement of the patterned surfaces of QD arrays, fluorescence microscopic images were collected. As presented in Figure 3a,b, when measured from the different locations (i.e., *X*_1_, *X*_2_, and *X*_3_ from the outermost to the innermost regions), the representative micrographs exhibit highly dense arrays of the ring-patterns with blue-emission from the arrays of the CsPbBr_3_ QDs. Although some undulations at the edges of the rings were observed, the pattern formation was successful on a large area without the use of lithographical templates or other expensive equipment. Notably, this one-step self-assembly process effectively rendered the perovskite QDs in the connected stripes by the cyclic stick-slip motions on a flat substrate, crafting the closely connected stripe configuration that has potential applications such as photodetectors or transistors in aligned arrays. Besides radially formed patterns at the three-phase contact line, dot arrays and spoke-like structures were also observed at the early stage of the solvent evaporation in some cases as evaluated by optical microscopic observation (Figure S4a). The spoke-like formation is attributed to the fingering instabilities at the drying front of the contact line as reported previously [26]. However, a structural transition was seen into ring patterns throughout the late evaporating process, when the meniscus gradually moves inward to the contact-center, as shown in Figure S4b. This characteristic feature of the assembly process may be due to the slightly large size distributions in the perovskite QD solution (Figure S1), which affect the speed of the solution front during the deposition process at the initial stage of the drying process. Thus, the interfacial interaction can be varied by the destabilization of the trapped solution. Indeed, by the careful preparation of the perovskite QD solution (i.e., higher-level centrifugation), as shown in Figure 3b, the finite aligned arrays of the stripes at the microscopic perspective depict a finely tuned controllability with high fidelity in the micro/nanoscale structures, facilitating a highly qualified colloidal QDs solution.

As mentioned earlier, the features of the rings appeared in a gradient fashion, representing the decrease in pattern distance between the neighboring rings as the cyclic stick-slip process at the edge of the meniscus approached the contact-center of the spherical lens/substrate. This result is mainly due to the curvature of the spherical lens placed on the flat substrate, which induces different height scales of the capillary bridge (i.e., evaporation rate). This geometrical factor governed the imbalance between the nonlinear capillary force and the linear pinning force; thus, the gradient patterns could be generated in the evaporating meniscus [31]. A recent report utilized these ring patterns consisting of perovskite QDs for a lasing source in an atmospheric environment, demonstrating superior optical gain properties [40]. In Figure 3c, a graph represents a high regularity of the position-dependent center-to-center distance (λ_c-c_) of the radially formed QD rings as a function of the *X* (i.e., distance from the contact-center). From the outermost to the innermost region, they were measured as λ_c-c_ = ~21.1 μm at *X* = ~3800 μm, λ_c-c_ = ~ 14.3 μm at *X* = ~3200 μm, λ_c-c_ = ~ 9.8 μm at *X* = ~2600 μm, and λ_c-c_ = ~5.8 μm at *X* = ~1800 μm, which revealed that the distance between the perovskite QD rings gradually decreased as they moved closer to the contact-center. This trend of pattern formation is similar to previous reports on the self-assembly of DNAs and polymeric solutions [41,42].

When a droplet of the CsPbI_3_ QD solution was engaged in the confined geometry, multiple concentric rings were produced. As presented in Figure 3d, the representative optical micrographs represent an array of the CsPbI_3_ QD rings with red-emission under the fluorescence. Interestingly, compared to the CsPbBr_3_ QD case, slightly different morphologies were found in the geometric configuration at the same concentrated solution (i.e., *c* = 0.2 mg mL^−1^). As shown in the inset (upper right), a phase-contrast optical micrograph indicates an array of sparsely discontinuous patterns in the formation of rings, which was not observed under fluorescence microscopy. Furthermore, from a macroscopic perspective, the radial shape was maintained but intermittently jammed QDs appeared in some locations regarding the movement of the solution front, as marked by yellow arrows (see a larger scale image in Figure S5). Here, the capillary-driven dragging force (i.e., perpendicular to the drying front of the contact line) and nucleation sites induced from the local roughness (i.e., radial pinning force) competed in the middle of the growth of the ring patterns [43]. We postulate that the precipitate of the unreacted slats on the prepared samples (i.e., CsPbI_3_ QDs) and the collection of the supernatant in the purification stage should be improved to reach better conditions in the self-assembly process. Because the surrounding QD solution between the two surfaces underwent constant pinning periods to form uniform arrays of rings, a more sophisticated microfluidic environment is required to extend our approach [33]. On the other hand, as shown in Figure 3e and Figure S6, the highly magnified optical micrographs reveal extremely ordered arrays of ring patterns in a gradient trend when measured at the outermost (upper) and innermost regions (lower).

**Figure 4:**
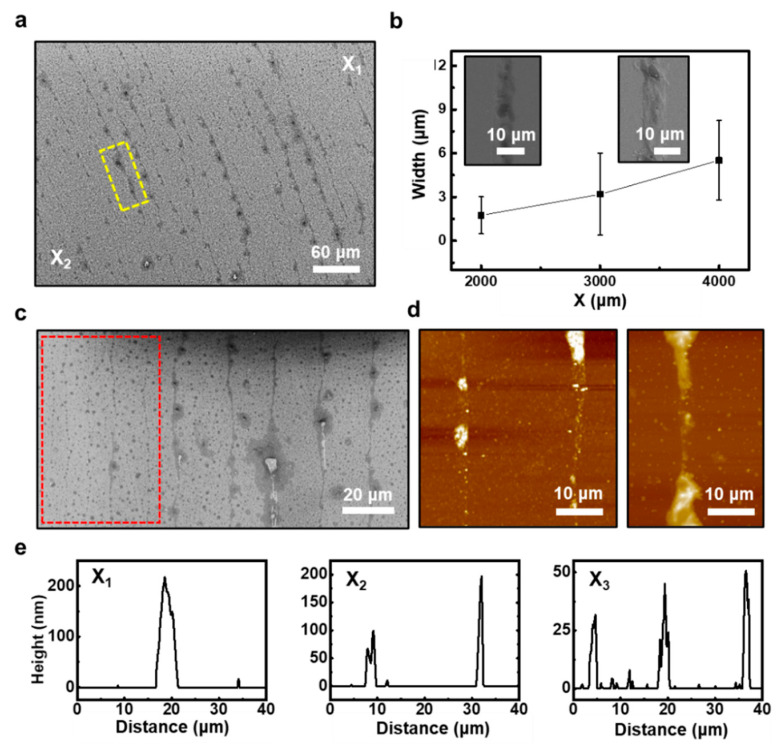
Dimensional configuration of the perovskite QD ring patterns. (**a**) SEM image of the perovskite QD multiple ring patterns. (**b**) The width of a single perovskite QD ring decreases as distance decreases from the contact-center. Inset SEM images show the width at 4000 μm and 2000 μm from the contact-center, respectively. (**c**) SEM image shows an array of narrow stripes in the innermost region that appeared with isolated QDs (the marked region with a dotted box). (**d**) Representative AFM images at the intermediate stage of the process; the magnified image of a single ring displays a nanostructured line induced from the weaker pinning force at the contact line in the deposition process. (**e**) The height transition of the multiple rings consisted of the perovskite QDs (i.e., CsPbBr_3_), depending on the ring position on the substrate from 200 nm at *X*_1_ to 25 nm at *X*_3_.

To explore the dimensional details of the perovskite QD ring patterns, SEM and AFM were used. Figure 4a shows a relatively regular formation of the discrete patterned array, and the gradient features were examined over the surface area. As presented in Figure 4b, the measured widths (*w*) of each ring extracted from the SEM images indicate a gradual decrease as the contact-center was approached. From the outermost to the innermost region, the range of the widths was found to be *w* = ~3.2 μm at *X* = ~4000 μm, *w* = ~2.9 μm at *X* = ~3000 μm, and *w* = ~1.6 μm at *X* = ~2000 μm; the representative magnified SEM images are shown in the insets. Surprisingly, by zooming in on the innermost region, an array of narrow rings appeared with isolated QDs in the marked region with a dotted box. Figure 4d shows a typical AFM imaged at the intermediate stage of the process, in which the magnified image of a single ring displays a nanostructured tiny line induced from the weaker pinning force at the contact line in the deposition period. This interesting morphology is similar to the molecularly combed DNA arrays with an aligned configuration produced from drying-mediated self-assembly, as reported previously [44]. By dimensional similarity, we may postulate that the small molecules can be assembled with the same strategy [45,46]. However, these slight variations in the fidelity and uniformity of the patterned configuration can be improved with a sufficient purification of QDs with a modified synthetic manner to extend our approach [38]. For more information, the height levels of each ring at the different regions were estimated by AFM measurement as shown in Figure 4e. The intrinsic curvature in the sphere-on-flat geometry dynamically generated the decreased height from ~200 to ~25 nm from the outermost to innermost locations through the recessive deposition of the QD solutes. As the evaporation progressed, the local concentration of QDs solution slowly decreased, and the width and height of the rings were thereby receded by propagating the solution inward. The above collective set of data revealed the geometric features of the concentric rings with exclusively patterned surfaces composed of the micro/nanostructured perovskite QDs.

## 4. Conclusions

In summary, we developed a unique, evaporative, self-assembly utilizing confined geometry (i.e., sphere-on-flat) to control the deposition process of the colloidal metal halide perovskite nanocrystals (i.e., CsPbBr_3_ and CsPbI_3_) dispersed in a volatile solvent. The optimized synthetic and patterning approach can simply and cheaply provide functional nanomaterials. As a result, guided by the upper spherical lens, the regularly ordered structures of concentric rings, composed of the perovskite QDs, were formed on a SiO_2_/Si or glass substrate. By the constrained evaporation rate of solvent, a drying-mediated robust assembly of the QDs was crafted within the control coverage range of millimeter-scale. The spatially well-arranged perovskite QD rings with high fidelity highlight the high yield strategy without the use of expensive equipment and a multi-step process with sacrificial layers for the affordable pattern transfer. The simplicity of our approach to organizing ‘coffee-rings’ of functional nanocrystals offers potential uses for applications in optoelectronic devices, such as LEDs, lasers, and photodetectors. Moreover, we envision that the material design and development will be extended to the metal halide perovskite-based optical sensing applications in a biological environment [47,48].

## Data Availability

The data presented in this study are available on request from the corresponding authors.

## Supplementary Materials

The following are available online at https://www.mdpi.com/article/10.3390/ma14226824/s1, Figure S1: Size distribution of (a) CsPbBr_3_ QDs and (b) CsPbI_3_ QDs; Figure S2: TEM image of CsPbBr_3_ QDs integration and morphology change without excess zinc salts; Figure S3: Control factors for highly ordered multiple ring pattern formation in sphere-on-flat geometry. Figure S4: (a) Optica; microscopic images show dot arrays and spoke-like structure formed at the early stage of the solvent evaporation. (b) A structural transition to the radially formed patterns of CsPbBr_3_ QDs at the three-phase contact line by consecutive stick-slip motion when the meniscus gradually moves inward to the contact center; Figure S5: The typical fluorescence micrographic image of CsPbI_3_ QDs rings with red emission, in which intermittently aggregated QDs in every single ring was observed on the scale of a millimeter; Figure S6: The digital images show blue emission and red-emission from the finite ring-patterned arrays of (a) CsPbBr_3_ and (b) CsPbI_3_ on the SiO_2_/Si flat surface under the excitation of a UV lamp (365 nm wavelength). (c) the λc-c identically decreases as toward the contact center when using the optimized colloidal concentration (c = 0.2 mg mL^−1^) of CsPbBr_3_ (blue dots) and CsPbI_3_ (red dots) QDs.
