# Supplementary material for: Spatially Ordered Arrays of Colloidal Inorganic Metal Halide Perovskite Nanocrystals via Controlled Droplet Evaporation in a Confined Geometry
Source: Materials (Basel). 2021 Nov 12;14(22):6824. doi: 10.3390/ma14226824 (PMC8618760; doi:10.3390/ma14226824)
Supplement: Supplementary file 1 [file materials-14-06824-s001.zip › materials-1436955-supplementary.pdf]

materials

Supplementary Materials

Spatially Ordered Arrays of Colloidal Inorganic Metal Halide Perovskite Nanocrystals via Controlled Droplet Evaporation in a Confined Geometry

Kwan Lee[1,*], Jonghyun Moon[2], Jeonghwa Jeong[2] and Suck Won Hong[2,*]

[1] Department of Advanced Materials Engineering, Kyungsung University, Busan 48434, Republic of Korea
[2] Department of Cogno-Mechatronics Engineering, Department of Optics and Mechatronics Engineering, Pusan National University, Busan 46241, Republic of Korea
* Correspondence: kwanlee@ks.ac.kr (K. L.); swhong@pusan.ac.kr (S. W. H.)

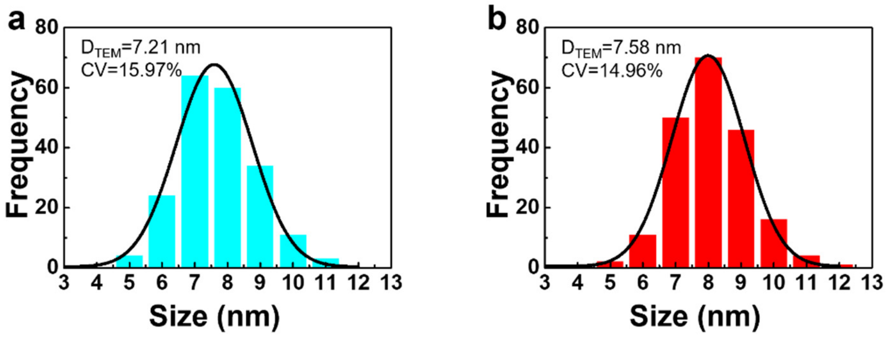

Figure S1. Size distribution of (a) $CsPbBr_3$ QDs and (b) $CsPbI_3$ QDs.

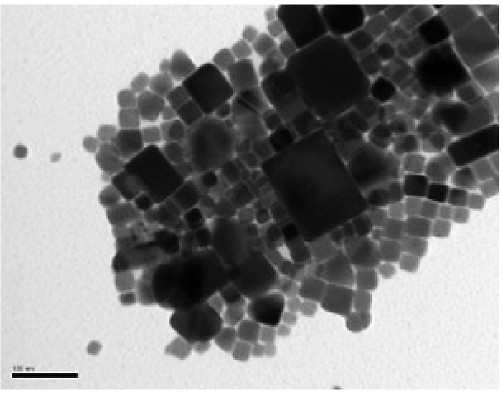

Figure S2. TEM image of CsPbBr3 QDs integration and morphology change without excess zinc salts.

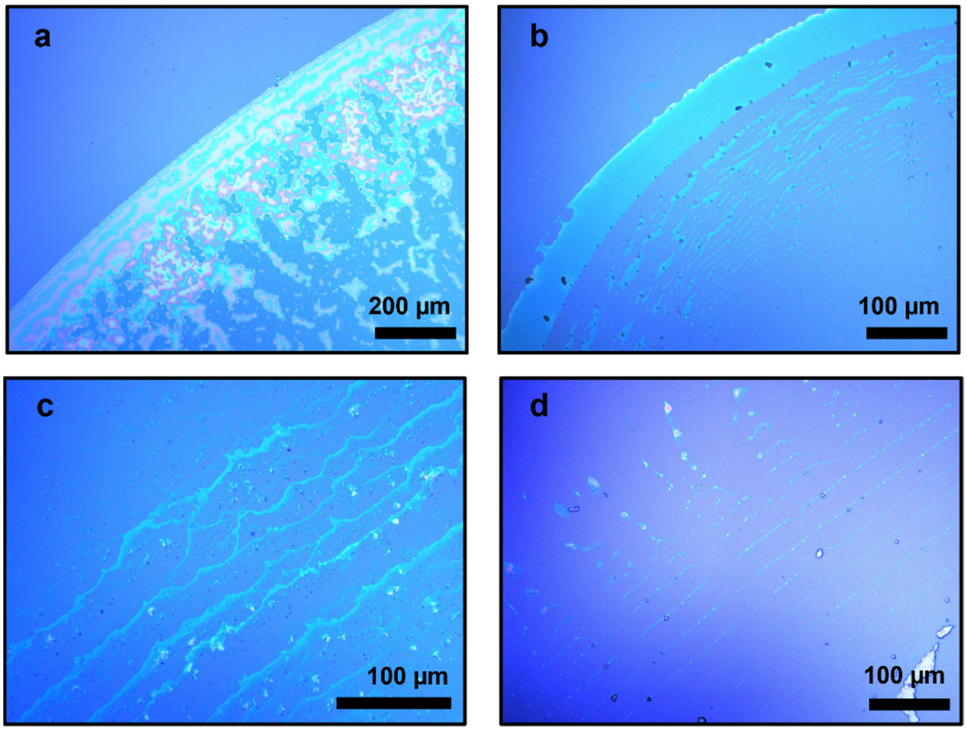

Figure S3. Control factors for highly ordered multiple ring pattern formation in sphere-on-flat geometry. (a) Poor purification for perovskite colloidal solution affects the delicate interaction within the two surfaces of the geometry. (b-c) Chaotic dissipated patterns were formed when the environmental temperature was higher than room temperature (i.e., ~60°C). (d) lower concentration than the optimized condition (c = 0.2 mg mL^{-1}) led to tiny lines but disconnected ring patterns with fingering instabilities.

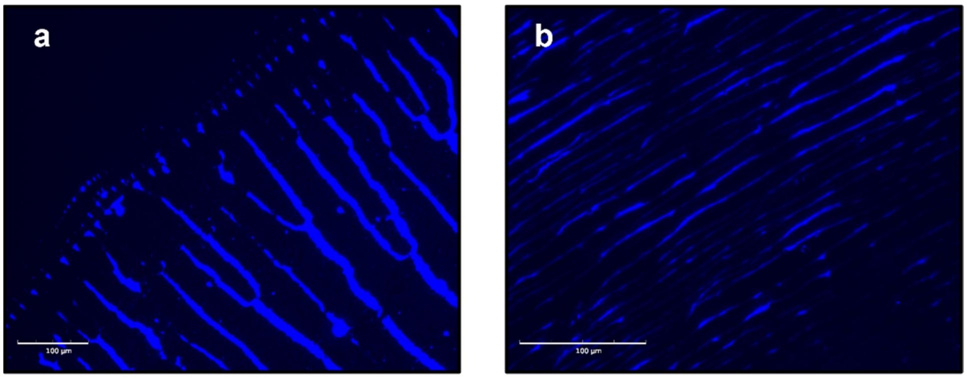

Figure S4. (a) Optica; microscopic images show dot arrays and spoke-like structure formed at the early stage of the solvent evaporation. (b) A structural transition to the radially formed patterns of $CsPbBr_3$ QDs at the three-phase contact line by consecutive stick-slip motion when the meniscus gradually moves inward to the contact center.

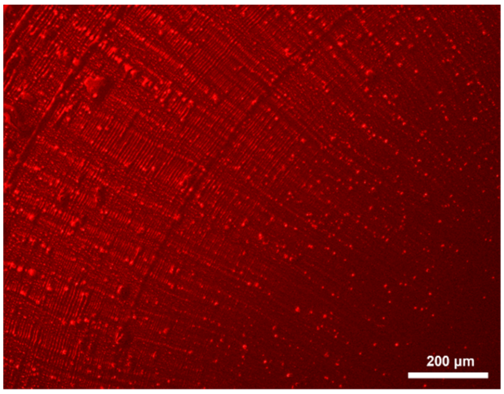

Figure S5. The typical fluorescence micrographic image of CsPbI3 QDs rings with red emission, in which intermittently aggregated QDs in every single ring was observed on the scale of a millimeter.

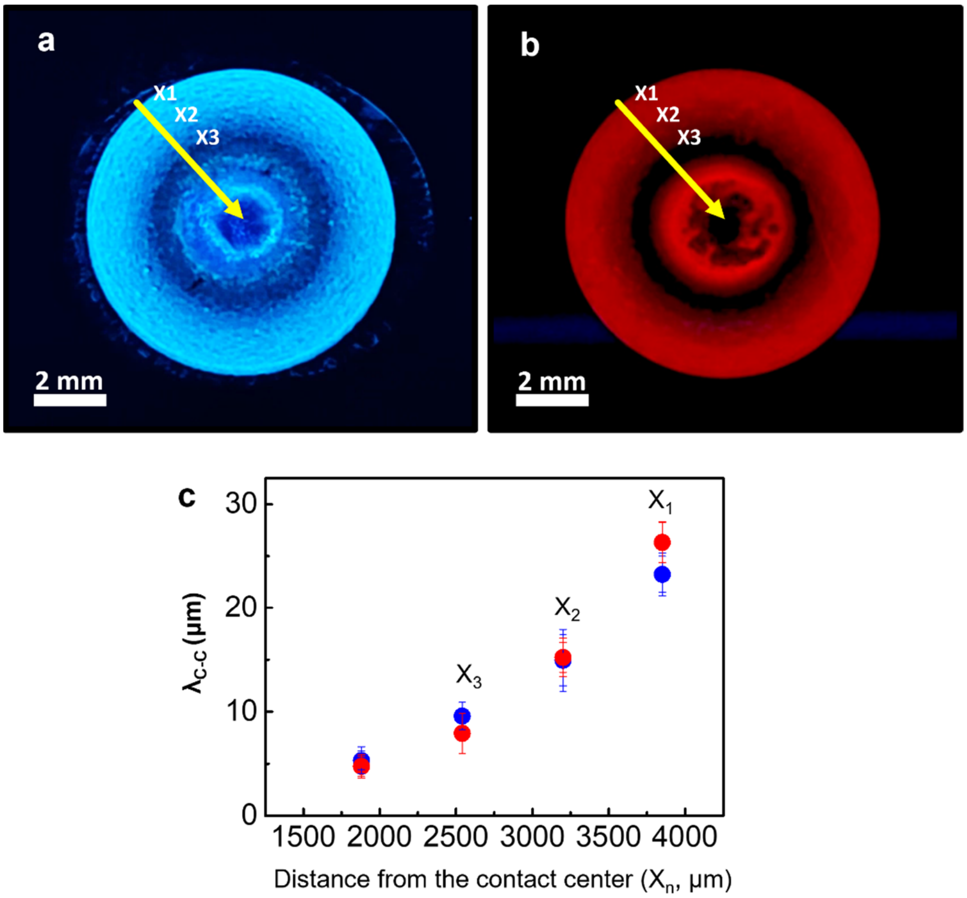

Figure S6. The digital images show blue emission and red-emission from the finite ring-patterned arrays of (a) $CsPbBr_3$ and (b) $CsPbI_3$ on the SiO_2/Si flat surface under the excitation of a UV lamp (365 nm wavelength). (c) the $\lambda_{c\text{-}c}$ identically decreases as toward the contact center when using the optimized colloidal concentration (c = 0.2 mg mL^{-1}) of $CsPbBr_3$ (blue dots) and $CsPbI_3$ (red dots) QDs.
